# Supplementary material for: Fast topographic optical imaging using encoded search focal scan
Source: Nat Commun. 2024 Mar 7;15:2065. doi: 10.1038/s41467-024-46267-y (PMC10920621; doi:10.1038/s41467-024-46267-y)
Supplement: Supplementary file 1 — Supplementary Information [file 41467_2024_46267_MOESM1_ESM.pdf]

Supplementary Information: Fast topographic optical imaging using Encoded Search Focal Scan

Narcís Vilar[1,2], Roger Artigas[1], Martí Duocastella[2], and Guillem Carles[1]

[1]Sensofar Tech S.L, Parc audiovisual de Catalunya, BV-1274 km 1, 08225 Terrassa, Spain.
[2]Department of Applied Physics, Universitat de Barcelona, C/Martí i Franquès 1, 08028 Barcelona, Spain.

Supplementary Notes

1 Robust axial localisation using Encoded Search Focal Scan 2
1.1 Use of periodic modulation 5
1.2 Suppressing 2π unwrapping errors 6
2 ESFS reconstruction algorithm 9
3 Sensitivity analysis and achievable range 9
4 Experimental prototypes 14
5 Supplementary references 18

Supplementary Figures

1 Illustration of plane encoding using different codes. 3
2 Comparison of sinusoidal and pulsed illumination 7
3 Illustration of the robust calculation of the step number d 9
4 Processing pipeline of the ESFS method 11
5 Classification accuracy in a binary step of the ESFS method 12
6 Assessment of the axial localisation precision 13
7 Calculated signal strength as a function of the measurement range . . 14
8 View and characterisation of the first ESFS prototype 15
9 Assessment of topographic resolution and system noise 16
10 View of the second ESFS prototype implemented in a microscope . . . 17

Supplementary Tables

1 Simulation parameters for the temporally modulated light 6
2 Algorithm for topography reconstruction using ESFS 10
3 Accuracy evaluation 17

1 Robust axial localisation using Encoded Search Focal Scan

As described in the main text, Encoded Search Focal Scan (ESFS) imaging constitutes a new paradigm for retrieving a topography map of a three-dimensional object by optical means, with a much higher speed than what can be achieved using conventional approaches. The technique is based on acquiring a set of images, each captured while the focal plane is continuously moved across the measurement axial range and the illumination is modulated.

The proposed method is based on two stages. In the first stage, the axial measurement range is divided into N steps, and we acquire a few images, each with an illumination sequence. Such illumination sequence consists in turning the illumination on and off in such a way that information from different regions within the measurement range is captured. For example, by turning the illumination on during the first half of the measurement range and off during the second half, it is possible to determine whether the axial location of the sample is in the first or second half. This is, however, only possible if we are able to calculate a quantity that is sensitive to the optical focus. We do this by detecting high spatial frequency features in the acquired focal-sweep image: if we detect such content, it can be deduced that the sample was in focus at an axial location with the illumination on. If we then acquire a second image with the illumination activated only during the first and third quarters of the measurement range, then, in combination with the previous image, we would be able to identify in which quarter the sample is located. And so on: by acquiring further images with illumination turned on and off at smaller cyclic steps, we can locate the sample within 2^n axial bins if we acquire n such images. This process amounts to performing an axial binary search at each pixel. **Supplementary Figure 1(a)** illustrates such search using a binary code. Each camera pixel is classified as "detected" (assign 1) or "not detected" (assign 0) for each subset of axial regions (i.e. for each image), which provides a binary code for each pixel. Such classification is performed by applying a threshold to the optically-sectioned images (see Eq. (3) of the main text). As an example, in the binary code illustrated in **Supplementary Figure 1(a)**, a pixel that responds as detected in the first image and not detected in the second and third images, will be assigned the binary code `100` and deduced to be localised in the axial plane number 4 out of the eight planes that the measurement range is divided in. In the second stage, higher localisation precision is achieved by capturing n_2 additional focal-sweep images while modulating the illumination over time (n_2 is typically 3 or 4). In the current implementation, we selected a periodic modulation, and each of the n_2 images was acquired with a different phase shift. By using the algorithms for phase-shifting interferometry, it is possible to extract the topography of the sample wrapped at the steps of our illumination cycle. The results obtained in the first stage

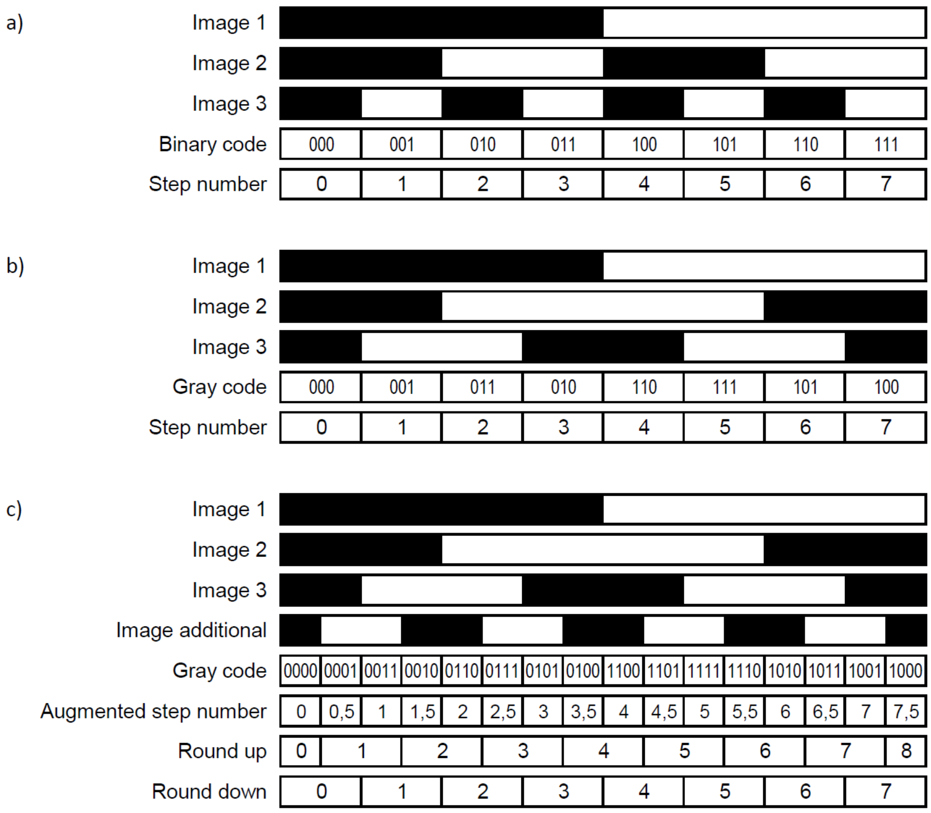

Supplementary Figure 1: (a) Illustration of a binary code for $n = 3$ bits and $2^n = 8$ axial planes. Each focal-sweep image provides a measurement of one bit, at each pixel. By acquiring n images, we can read the binary code associated with each pixel, and therefore its associated axial plane. (b) Illustration of plane encoding using a Gray code with $n = 3$ bits. (c) Illustration of plane encoding using a Gray code with 4 bits (augmented using the additional image). The augmented step number (divided by two to preserve the range) encodes the measurement range steps with double resolution, and by rounding up and down the augmented step number we obtain two measurements of the step number that are horizontally shifted.

can then be used to unambiguously unwrap this information and restore a topography map with high localisation precision and over a large axial range while using only $n + n_2$ images.

Of course, detection of the code from noisy measurements is subject to errors, specially if the object is located at an axial position near the transition, where the change of the illumination (from on to off, or *vice versa*) happens within the depth of field of the microscope objective. In such cases, the reading of the corresponding bit is corrupted by noise, resulting in visible artefacts in the reconstructed topography. Additionally, we use the results of the first stage of ESFS to unwrap the topography that is obtained in the second stage, and so such bit transitions coincide with the wrapping steps, which also produces visible artefacts, known as 2π errors. Here we describe the details of the implemented method to suppress these errors.

Using a binary code is helpful to understand the proposed technique, but it has a disadvantage: those transition errors correspond to axial locations that are coincident in different images, and therefore the impact of such errors in the determination of the axial plane can be very high. For example, in **Supplementary Figure 1(a)** an axial location close to the transition between step number 1 and 2 can have bits 2 and 3 erroneous, and so the reading could be anywhere between steps 0 and 3 inclusive. To solve this issue, a different coding sequence should be used. For example, using a Gray code [1] solves the issue, as is illustrated in **Supplementary Figure 1(b)**. As can be observed, it is possible to encode 8 planes and avoid simultaneous transitions in the different images/bits. In this case, as the axial position of the object is moved across the measurement range, only one bit is changed from one step to the next, as is expected from the design of the Gray encoding. Such encoding reduces the impact of detection errors to only one plane [1]. In the example of an axial location between steps 1 and 2, only bit 2 can be erroneous, and the encoding would provide either step 1 or 2.

A second source of reading error is the choice of the threshold value, $S_{\text{th}}(x,y)$ in Eq. (3) of the main text. Since the optimum value of the threshold depends on the reflectivity of the sample, it is not straightforward to determine the threshold for all field positions. To determine such a field-dependent threshold, we compute the intensity image as if it was acquired with the illumination turned constantly on during exposure. Such an image can be regarded as a normalisation image and used to null the effect of the sample reflectivity. Exploiting the fact that the acquired images in the second stage are phase shifted with n_2 equally-distributed phase shifts, we can estimate this signal map by,

$$S_{\text{th}}(x,y) = \frac{1}{n_2}\sum_{j=1}^{n_2} S_j(x,y) \tag{1}$$

$$= \frac{S_{\text{max}}}{2} \tag{2}$$

Equation (2) only holds if the illumination is exactly sinusoidal. However, since its use is to determine a threshold for classification, it can tolerate deviations and, in practice, Equation (1) can also be used using pulsed illumination (as described below). Alternatively, a straightforward and robust solution to determine $S_{\text{th}}(x,y)$ is to directly acquire an additional image with the illumination constantly on during exposure. Note that, besides increasing the total number of acquired images, with the consequent reduction in imaging speed, this additional image collects approximately twice the energy than images with Gray coded illumination. Therefore, to avoid sensor saturation, either the illumination intensity or the exposure time should be reduced. The latter can be achieved by axially scanning with double speed. In any

case, processing such constant-illumination image with exact operations and dividing by two, directly provides a sensible field-dependent value for the threshold $S_{\mathrm{th}}(x, y)$.

1.1 Use of periodic modulation

In the second stage of ESFS, in which the illumination intensity is periodically modulated in time, we intend to determine the axial location of the object with a precision higher than the size of the axial step. To this end, we determine the axial location as a phase measurement between axial steps, thereby obtaining the axial location of the sample modulo the step size. Since in the first stage we determine the step number of each pixel, we can unambiguously unwrap the measurement. As an example, we encode the axial location using a sinusoidally-modulated illumination in time while the sample is moved across the measurement range, as is described in the main text (see Eq. (6) that describes the particular case of using 3 measurements). In practice, a more robust measurement is obtained using a 4-step measurement with $\pi/2$ relative phase shift [2]. This is,

$$M_j(z) = \frac{1 + \cos\left(\frac{2\pi}{T}z + \delta_j\right)}{2} \qquad \text{for } \delta_j = \left\{-\pi, -\tfrac{\pi}{2}, 0, \tfrac{\pi}{2}\right\}. \tag{3}$$

where T is the size of the axial step. From the images acquired with such sequence and processed to compute the optically-sectioned signal we obtain,

$$S_1 = A - B\cos\left(\frac{2\pi}{T}z\right) \tag{4}$$

$$S_2 = A + B\sin\left(\frac{2\pi}{T}z\right) \tag{5}$$

$$S_3 = A + B\cos\left(\frac{2\pi}{T}z\right) \tag{6}$$

$$S_4 = A - B\sin\left(\frac{2\pi}{T}z\right) \tag{7}$$

where $A = S_{\max}/2$ and $B = mS_{\max}/2$, with m being the resulting reduction in contrast that appears due to the depth of field of the imaging system. The reconstructed topography modulo T can be readily calculated from,

$$\tan\left(\frac{2\pi}{T}z\right) = \frac{S_2 - S_4}{S_3 - S_1} \tag{8}$$

as,

$$\widehat{z_s}(x, y) = \frac{T}{2\pi}\arg\left((S_2 - S_4) + \imath\,(S_3 - S_1)\right) \tag{9}$$

where $\arg(z)$ is the argument of the complex number $z = x + \imath y$ in the range $[0, 2\pi)$.

It can be seen from Eq. (1) of the main text that the output signal measured at the image plane via optical sectioning, is the result of a convolution with an axial impulse function that depends on the depth of field of the objective employed. In practice, the effect of such convolution is to reduce the contrast of the detected signal, i.e. $m < 1$. On the other hand, employing sinusoidally-modulated illumination ensures that, regardless of contrast and the shape of the axial impulse function, the detected signal is also sinusoidal, and so Equations (4-9) are valid.

However, it is possible to increase contrast to some extent by using pulsed illumination. In this case, it can no longer be assumed that the measured signal is exactly sinusoidal, deviating from Equations (4-7), although such deviations may be accounted for through simple calibration. In particular, the use of pulsed illumination has the additional advantage of being easier to implement, as there is no need for precisely varying the illumination intensity. Furthermore, if the illumination period is not far from the depth of field of the objective, then the difference from the expected sinusoidal illumination is small, as higher harmonics are attenuated. This is due in part to the finite response time of the light source but, mostly, to the axial convolution with the 3D point-spread function of the instrument. In practice, illumination with a train of pulses and direct application of Equations (4-9) provides a similar performance to implementing illumination with sinusoidally-modulated intensity. To illustrate and quantify this effect, simulation experiments are shown in **Supplementary Figure 2** comparing pulsed and sinusoidal illumination (simulation parameters shown in **Supplementary Table 1**).

Supplementary Table 1: Simulation parameters to compare axial localisation precision using sinusoidally modulated light with pulsed light.

Parameter	Value
Magnification	20×
Numerical aperture	0.45
Wavelength	0.5 µm
Step size T	10 µm
Illumination pattern	Checkerboard
Pattern pitch	17.25 µm
σ_1	2 px
σ_2	5 px
Pixel size	3.45 µm

1.2 Suppressing 2π unwrapping errors

Since the reconstruction of the topography using Eq. (9) provides a phase measurement, it results in a wrapped output, providing the topography of the sample modulo the distance that corresponds with the period of the modulation, T. For error-free unwrapping, the axial locations where the axial wrapping occurs should precisely co-

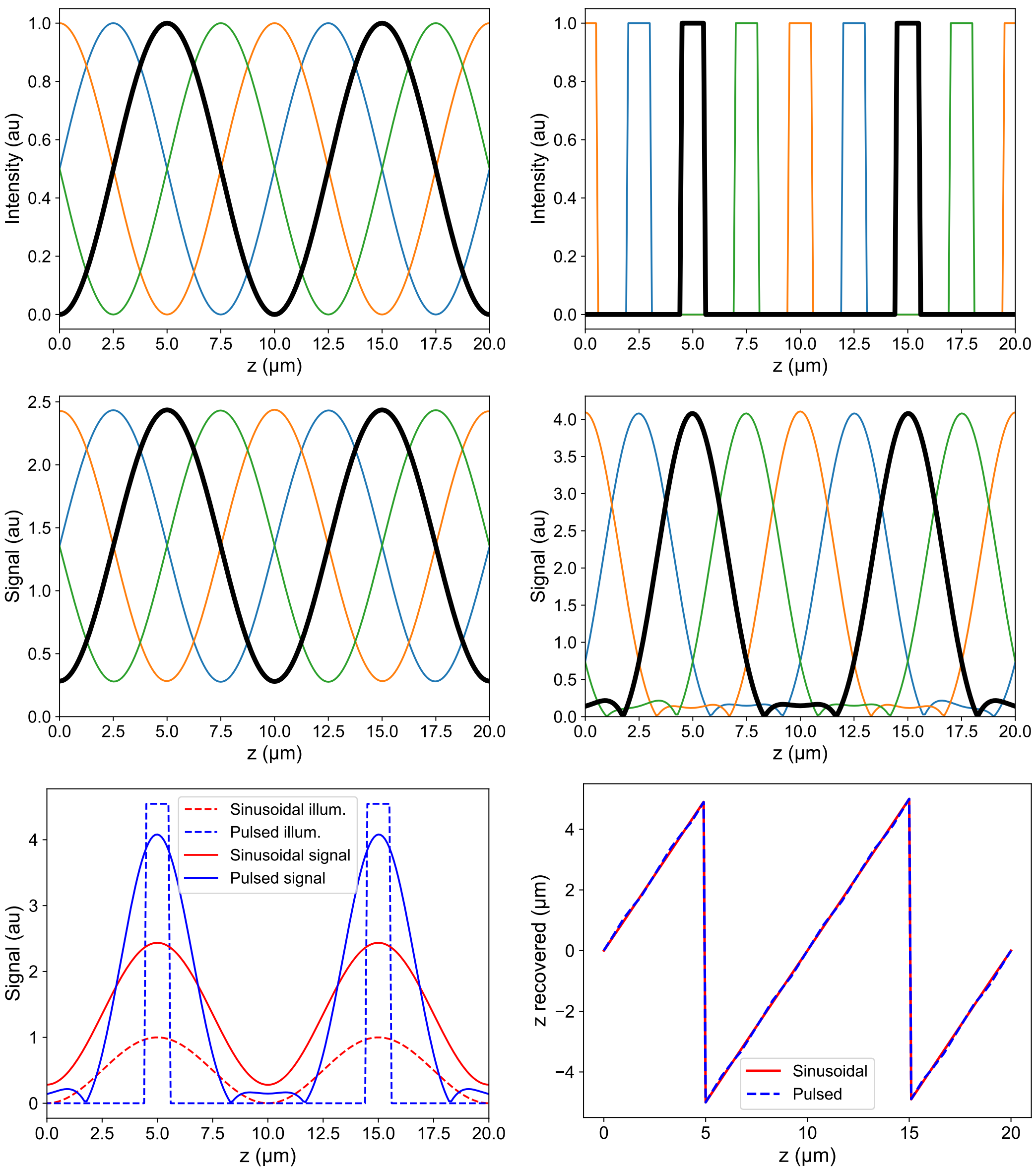

Supplementary Figure 2: Comparison of sinusoidal and pulsed illumination. In the first row are shown sinusoidal (left) and pulsed (right) illumination. The period is set to $T = 10\,\mu\text{m}$, and the illumination is plotted for four time delays corresponding to $\Delta z = T/4$, in different colours. In the second row, it is plotted the simulated acquired signal (Eq. (1) of the main paper), again for sinusoidal (left) and pulsed (right) illumination. For comparison, both sinusoidal and pulsed cases are plotted together in the bottom left graph, where the difference in signal contrast is evident (note pulsed illumination was scaled to provide the same integrated image intensity). Comparison of results using Eq. (6) of the main paper (which assumes signals are sinusoidal) are plotted in the bottom right graph, showing very small differences even though the deviation of the pulsed-illumination signal differs significantly from the sinusoidal form.

incide with the bit transitions of the Gray code. However, imperfect synchronisation and also noise in the measurements prevent this ideal situation, yielding 2π errors in the reconstruction. To solve this issue, we use an additional Gray code bit (i.e. an additional image) as was proposed in the field of fringe projection [3]. Using such an additional image, the measurement range can be divided into twice the number of original steps, that is, 2^{n+1} steps, whose step number is represented by d_{n+1} (see **Supplementary Figure 1(c)**). By defining an augmented step number $d_+ = d_{n+1}/2$, we can calculate the step number d as,

$$d = \begin{cases} \lceil d_+ \rceil & \text{if} \quad 0 \leq \widehat{z_s}(x,y) < \frac{1}{4}T \\ \lfloor d_+ \rfloor & \text{if} \quad \frac{1}{4}T \leq \widehat{z_s}(x,y) < \frac{3}{4}T \\ \lceil d_+ \rceil - 1 & \text{if} \quad \frac{3}{4}T \leq \widehat{z_s}(x,y) < T \end{cases} \tag{10}$$

where the operators $\lceil \ \rceil$ and $\lfloor \ \rfloor$ stand for rounding up and down to the nearest integer respectively. Note that the calculation of the augmented step number performed using the additional image results in a discretisation of the measurement range into $2N$ steps of size $T/2$. By rounding the augmented step number up and down, these $2N$ steps can be reduced to two discretisation sets, each containing N steps occurring at alternating locations (see **Supplementary Figure 1(c)**). The key to avoiding 2π errors is to use the phase information in $\widehat{z_s}(x,y)$ to conveniently select a discretisation set that has no bit transitions in the given axial location. This process is illustrated in **Supplementary Figure 3**. The final values of d are coincident with $\lfloor d_+ \rfloor$. However, for its determination from noisy data, information is taken from redundant data in such a way that bit transitions are avoided completely. As is shown in the close-up view in **Supplementary Figure 3**, in the central region of the cycle (green arrow region) $\lfloor d_+ \rfloor$ is used, whereas in the outer regions (red arrows region) $\lceil d_+ \rceil$ is used; and there are no bit transitions in either case. Therefore, such an approach enables 2π errors to be virtually eliminated.

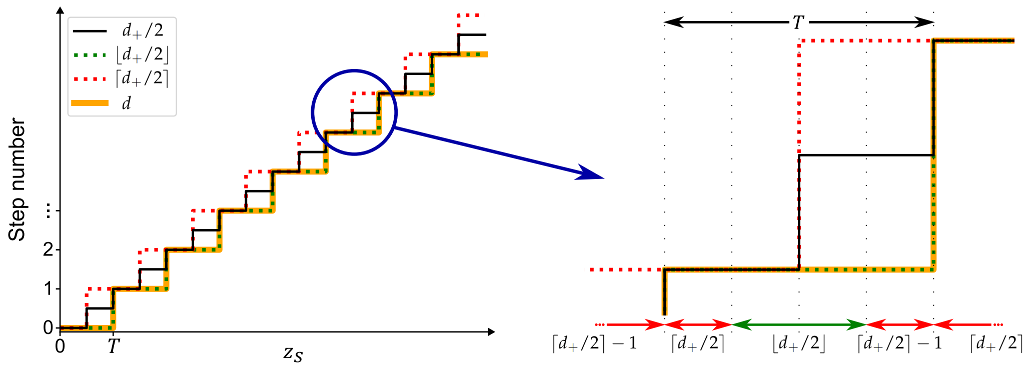

Supplementary Figure 3: Illustration of the robust calculation of the step number d. A sequence encoded with a Gray code using $2N$ steps is implemented and the resulting step number d_+ is plotted with the black continuous line (divided by 2 to match the N-step range). By rounding this quantity up and down we obtain two copies of the N-step encoding sequence horizontally shifted by half step, which are plotted with the dotted lines in red and green, respectively. The final calculation of the step number, d, is extracted in a piece-wise manner from the latter two, as in Eq. (10), and is used for the unwrapping of $\widehat{z}_S$. The result is plotted with a thick orange line. This piece-wise reconstruction is illustrated in the close-up view shown at the right: within the second and third quarters of the step, information from the rounding-down (green) function is used, whereas in the first and fourth quarters, information from the rounding-up (red) function is used, as illustrated at the bottom of the close-up view. This ensures d is computed from data without step transitions.

2 ESFS reconstruction algorithm

The algorithm for the reconstruction of the topography using ESFS is shown in **Supplementary Table 1**, using $n+1$ focal-sweep images in the first stage and 4 images in the second stage (as described in the previous supplementary notes).

An illustration of the processing pipeline is shown in **Supplementary Figure 4** using the reconstruction of a topography of a tilted mirror from experimental data acquired with the first prototype.

3 Sensitivity analysis and achievable range

As discussed, the ESFS method provides orders of magnitude speed enhancements compared to traditional approaches, because the number of images that need to be acquired is reduced dramatically. It may seem natural to ask if the price to pay is a reduced precision in the measurement. On one hand, the integration through the entire measurement range reduces the dynamic range of the ESFS images, effectively yielding a lower signal-to-noise ratio. Note that such a reduction occurs if the focus-sensitive signal is computed in a post-detection step, which would not be the case if a focus-sensitive signal, such as a confocal signal, is directly acquired. On the other hand, in the traditional approaches, a high amount of data is collected that carries

Supplementary Table 2: Topography reconstruction using ESFS

Algorithm 1: Topography reconstruction using ESFS with n images in the first stage and $n_2 = 4$ images in the second stage

---

Input: $\{I_i^{(1st)}(x,y)\}$, a set of $n+1$ focal-sweep images obtained with synchronised illumination implementing a Gray code as in **Supplementary Figure 1(c)**;

GC, a look-up-table with 2^{n+1} entries relating a binary number to the Gray code; and

$\{I_i^{(2nd)}(x,y)\}$, a set of 4 focal-sweep images obtained with periodic illumination, each subsequently delayed by a forth of the period.

Output: $z_s(x,y)$, the reconstructed topographic height map of the sample.

// Apply optical-sectioning operator (OS) to all input images
for $i = 1, \ldots, (n+1)$ **do**
    forall (x,y) **do**
        $S_i^{(1st)}(x,y) \longleftarrow \mathrm{OS}\left[I_i^{(1st)}(x,y)\right]$ // OS operator as defined in Equation (2)

for $j = 1, \ldots, 4$ **do**
    forall (x,y) **do**
        $S_j^{(2nd)}(x,y) \longleftarrow \mathrm{OS}\left[I_j^{(2nd)}(x,y)\right]$

// Calculate the wrapped topography
forall (x,y) **do**
    $I_{\mathrm{top}} \longleftarrow S_2^{(2nd)}(x,y) - S_4^{(2nd)}(x,y)$
    $I_{\mathrm{bot}} \longleftarrow S_3^{(2nd)}(x,y) - S_1^{(2nd)}(x,y)$
    $\widehat{z}_s(x,y) \longleftarrow \mathrm{atan2}(I_{\mathrm{top}}, I_{\mathrm{bot}}) \cdot T/(2\pi)$ // quadrant-sensitive arctangent function

// Calculate field-dependent threshold, Equation (S1)
forall (x,y) **do**
    $S_{\mathrm{th}}(x,y) \longleftarrow \left(S_1^{(2nd)}(x,y) + S_2^{(2nd)}(x,y) + S_3^{(2nd)}(x,y) + S_4^{(2nd)}(x,y)\right)/4$

// Obtain the augmented step number
forall (x,y) **do**
    $d_{n+1}(x,y) \longleftarrow 0$

for $i = 1, \ldots, (n+1)$ **do**
    foreach (x,y) *such that* $S_i(x,y) > S_{\mathrm{th}}(x,y)$ **do**
    $d_{n+1}(x,y) \longleftarrow d_{n+1}(x,y) + 2^{n+1-i}$

forall (x,y) **do**
    $d_+(x,y) \longleftarrow \mathrm{GC}\left[d_+(x,y)\right]/2$ // Apply look-up-table for decoding

// Calculate the step number
forall (x,y) **do**
    if $\widehat{z}_s(x,y) < T/4$ **then**
        $d(x,y) \longleftarrow \mathrm{ceil}\left[d_+(x,y)\right]$ // ceil is round-up to the nearest integer
    else if $T/4 \leq \widehat{z}_s(x,y) < 3T/4$ **then**
        $d(x,y) \longleftarrow \mathrm{floor}\left[d_+(x,y)\right]$ // floor is round-down to the nearest integer
    else
        $d(x,y) \longleftarrow \mathrm{ceil}[d_+(x,y)] - 1$

// Calculate output topography
forall (x,y) **do**
    $z_s(x,y) \longleftarrow d(x,y) \cdot T + \widehat{z}_s(x,y)$

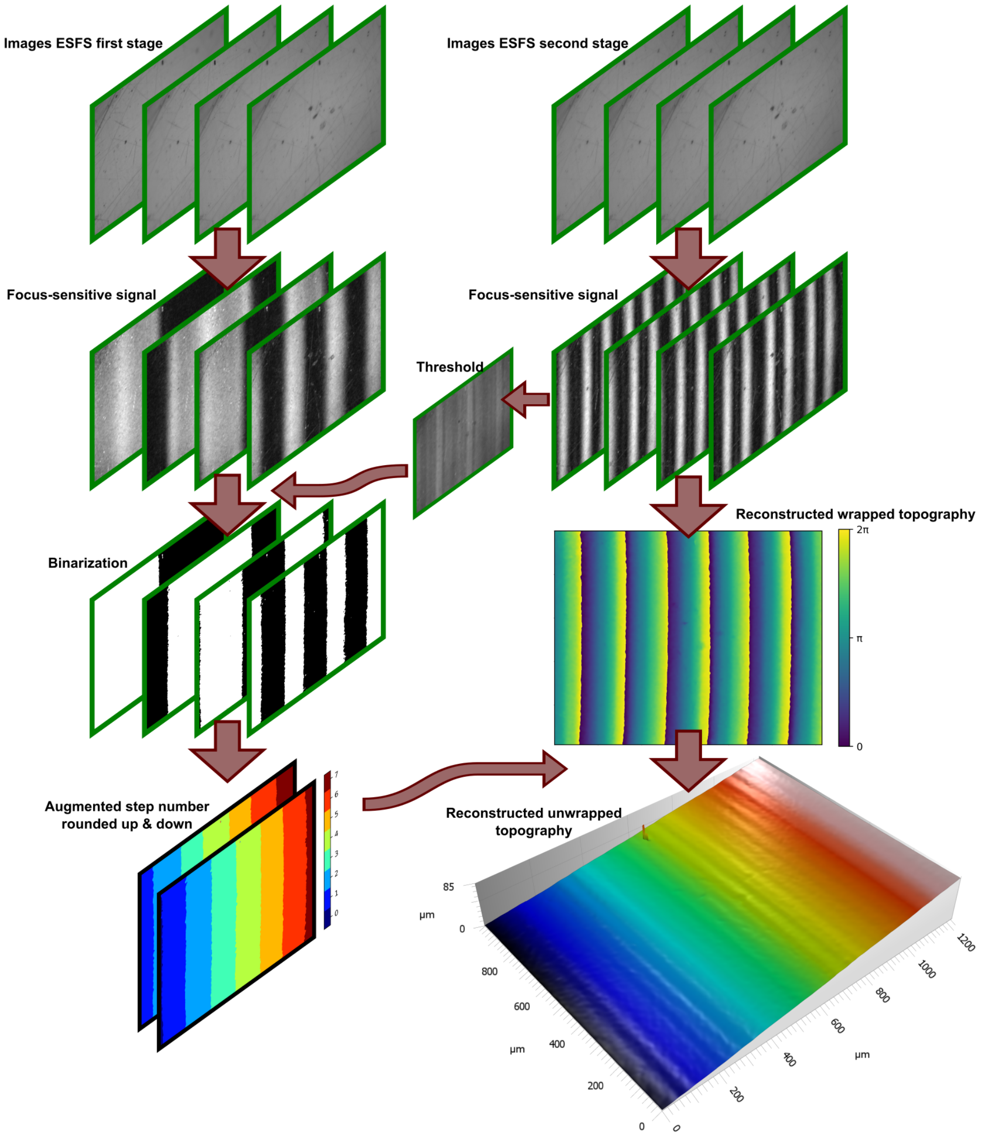

Supplementary Figure 4: Processing pipeline of the ESFS method for the reconstruction of the topography of a tilted mirror. Eight input images are shown at the top, four for the first stage (left) and four for the second stage (right). From these images, calculation of the focus-sensitive signal is computed using Equation (2) of the main text, and the resultant images are shown in the second row. For the first stage (left), focus-sensitive images are binarised and a coarse/discretised topography map is calculated. For the second stage (right), the phase-shifted focus-sensitive signals are used to calculate the more precise but wrapped topography of the sample. Finally, the coarse and wrapped components are combined to yield the output topography (bottom-right).

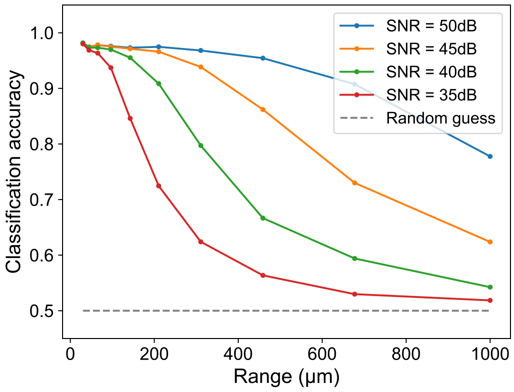

Supplementary Figure 5: Classification accuracy in a binary step of the ESFS method. The step size is set to $T = 10\,\mu m$, imaging NA is 0.45, and a range of detector noise is considered. Values of SNR in the legend stand for signal-to-noise ratio of the intensity values of the input images, i.e. detector noise. It can be seen how, by increasing the measurement range, the classification accuracy deteriorates but remains at useful levels for even large ranges.

no meaningful information. It is precisely these data that ESFS avoids collecting. To quantify this, we show here three different simulation experiments.

First, we consider the accuracy in detecting the sample from one image in the first stage of the ESFS method as a function of the noise level in the acquired image. Such detection shall be considered a binary classification problem, as the image (or more precisely the region around a pixel in the image) is classified as "sample present" or "sample not present" by the application of a threshold on the calculated focus-sensitive signal. To estimate such classification accuracy, we simulate image acquisition including detector noise, calculate the optical sectioning signal, and use its binarisation to determine the presence or absence of the sample (Eqs. (1-3) of the main paper). By repeating the process with the sample located at random positions within the measurement range, we assess the classification accuracy as a function of the measurement range and noise level. Results are shown in **Supplementary Figure 5**, where they are observed to follow the expected trend, as increasing the measurement range decreases the contrast of the signal, thereby reducing the signal-to-noise ratio. These results indicate that the binary stage of the ESFS method is useful even for very large measurement ranges (depending on camera noise).

Secondly, we explore the localisation precision of the second stage of the ESFS method. To do this, we simulate image acquisition (including detector noise), calculate the optical sectioning signal, perform axial localisation using Eq. (9), and calculate the localisation error. Repeating the process for a range of random axial locations we obtain the simulated precision of the method. Results are shown in **Supplementary Figure 6**, as a function of the measurement range for a fixed step size of $T = 10\,\mu m$ (left) and

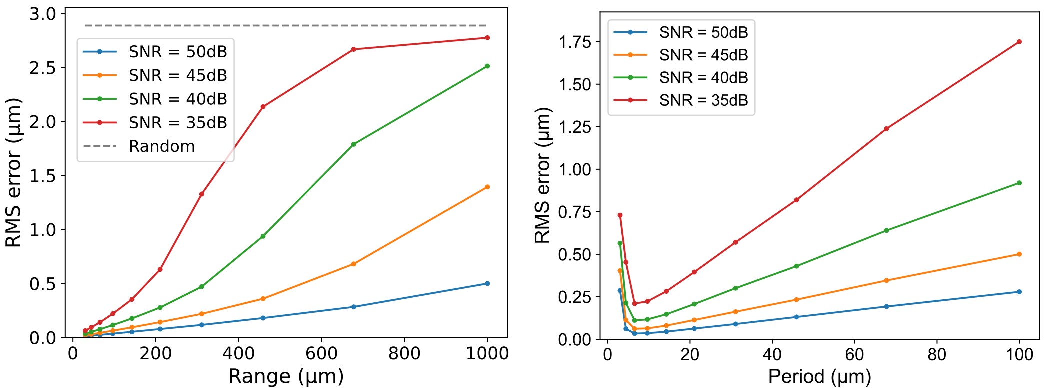

Supplementary Figure 6: Assessment of the axial localisation precision. Simulated experiments are repeated with true sample locations randomly distributed within the step size T. Results for $T = 10\,\mu\text{m}$ are shown in the left graph for different measurement ranges. Because $T = 10\,\mu\text{m}$, a random estimator would statistically result in a precision of $T/\sqrt{12} = 2.89\,\mu\text{m}$. Results for a 100 μm measurement range and for different values of the period T are shown in the right graph. Simulation results use an imaging NA of 0.45.

as a function of the step size T for a fixed range of 100 μm (right). It can be seen how, for a fixed step size, increasing the measurement range leads to the deterioration in precision due to the contrast reduction of the acquired images. Instead, for a fixed range, there is an optimal localisation precision at the vicinity of the depth of field (DOF) of the objective. For a step size smaller than the DOF, precision drops due to the degeneration of data. For values larger than the DOF, the localisation precision worsens linearly with the step size T.

Finally, we explicitly calculate the signal-to-noise ratio of our focus-sensitive signal S. The strength of the signal computed with and without the projected pattern is calculated to assess the strength against noise. Results are shown in **Supplementary Figure 7** for a range of detector noise levels. As expected, the SNR decreases with the axial scanned range for all conditions analysed. Note that a significant SNR value remains even for very large ranges, as is also observed in **Supplementary Figure 6(a)**.

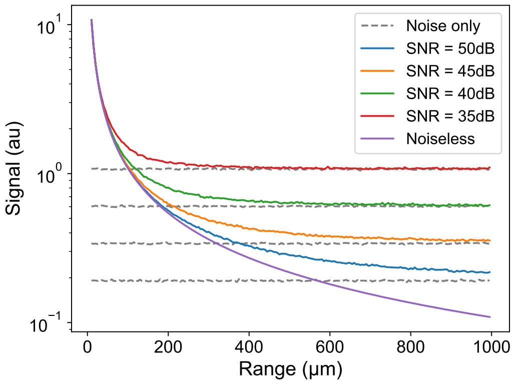

Supplementary Figure 7: Calculated signal strength as a function of the measurement range for a different detector noise levels.

4 Experimental prototypes

We reported two implementations of the ESFS method, and we provide here further details and characterisation of the systems. The first implementation is based on a motorised stage and is shown in **Supplementary Figure 8(a)**. To assess the performance of the system, we measured a step height sample with a certified value of 21.702 µm ± 0.022 µm, and a tilted mirror. Measurements are shown in **Supplementary Figure 8(b,c)**. The measured value of the step height was 21.704 µm which is in excellent agreement with the certified value. We fitted a plane to the topographic measurement of the tilted mirror and calculated the root mean squared error of the residuals, which provided a value of $S_q = 0.59$ µm. Note that this quantity is affected by measurement noise but mainly by systematic non-linearity errors, as indicated by the ripples of the residual errors. Finally, an assessment of the system (repeatability) noise was performed by sequentially acquiring two measurements of a flat mirror and calculating the differences (and dividing by $\sqrt{2}$ to account for the fact that noise is present in both acquisitions), which resulted in a root mean squared value of,

$$E_{\text{noise}} = 0.045\,\mu\text{m} \tag{11}$$

Of course, the noise level is dependent on the size of kernel filters applied (Equation (2) of the main text), which in this case we set to $\sigma_b = 5$ px as higher values would decrease the lateral resolution.

To assess the lateral resolution of the system, and to compare it with a conventional method for reference, we measured a topographic Siemens star target, as shown in **Supplementary Figure 9**. The target consists of 18 pairs of circular sectors with a nominal height of 0.186 µm. We performed two different sets of measurements both using a 50×/0.8NA objective and a total measurement range of 15 µm. The first one

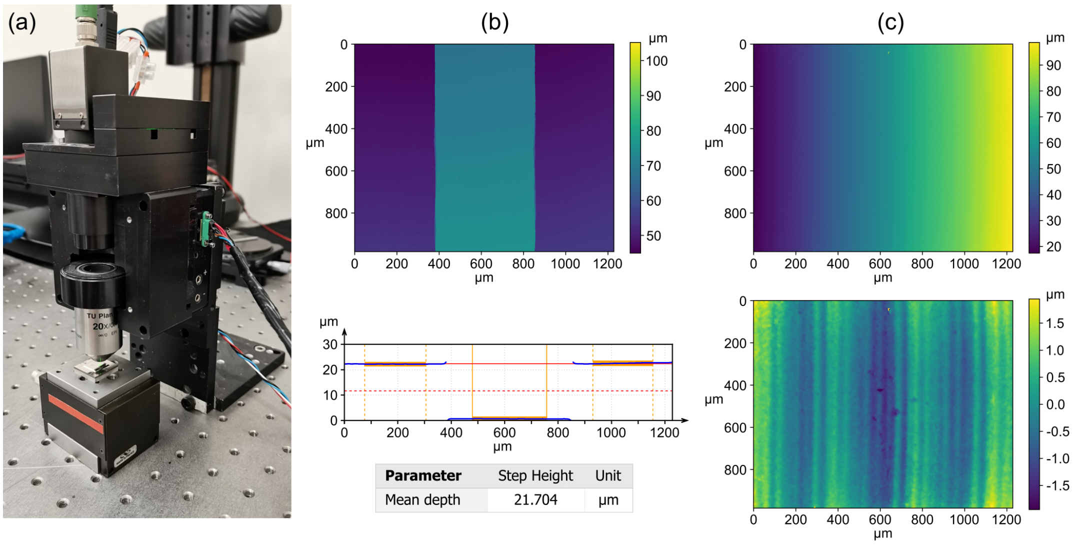

Supplementary Figure 8: View and characterisation of the first prototype. A photograph of the system is shown in (a). Measurement of a step height specimen is shown in (b) with a profile plot at the bottom graph. Measurement of a tilted mirror is shown in (c) with a plot of the residual error from the fitting of a plane shown at the bottom.

with ESFS and the second with a conventional technique for comparison. The selected technique is "focus variation with structured illumination" as it provides a fair comparison with our implementation of ESFS. For each case, the measured height was calculated by analysing the contrast of concentric circular profiles. Each profile (at varying radial distances) samples the modulated height of the target at a different spatial frequency. This enables to compute the Instrument Transfer Function (ITF) of the system, a quantity that relates the ratio between the measured to the nominal height as a function of the spatial frequency. The cut-off frequency of the ITF is therefore an indication of the topographic resolution of the system. As shown in **Supplementary Figure 9(b)**, the calculated ITF in the case of the conventional method reaches higher spatial frequencies than ESFS. This indicates a loss of spatial resolution in the latter, arguably caused by the lower SNR in the focal-sweep images.

Given that the current ESFS implementation is based on focus variation, the reported lateral resolution values depend on the kernel filters applied (σ_b in the outer filter in Equation (2)). Increasing σ_b reduces the lateral resolution, but a minimum value of this parameter is necessary to calculate the optical section image needed for topography reconstruction. Instead, and as stated before, the system noise decreases with σ_b. To experimentally quantify this trade-off for the case of ESFS and also conventional focus varation, we calculated the loss in lateral resolution and the reduction in system noise as a function of σ_b. The resolution was estimated as the inverse of the spatial frequency at which the ITF falls at 10%, and the system noise was computed by subtracting two consecutive measurements divided by $\sqrt{2}$. The results shown in

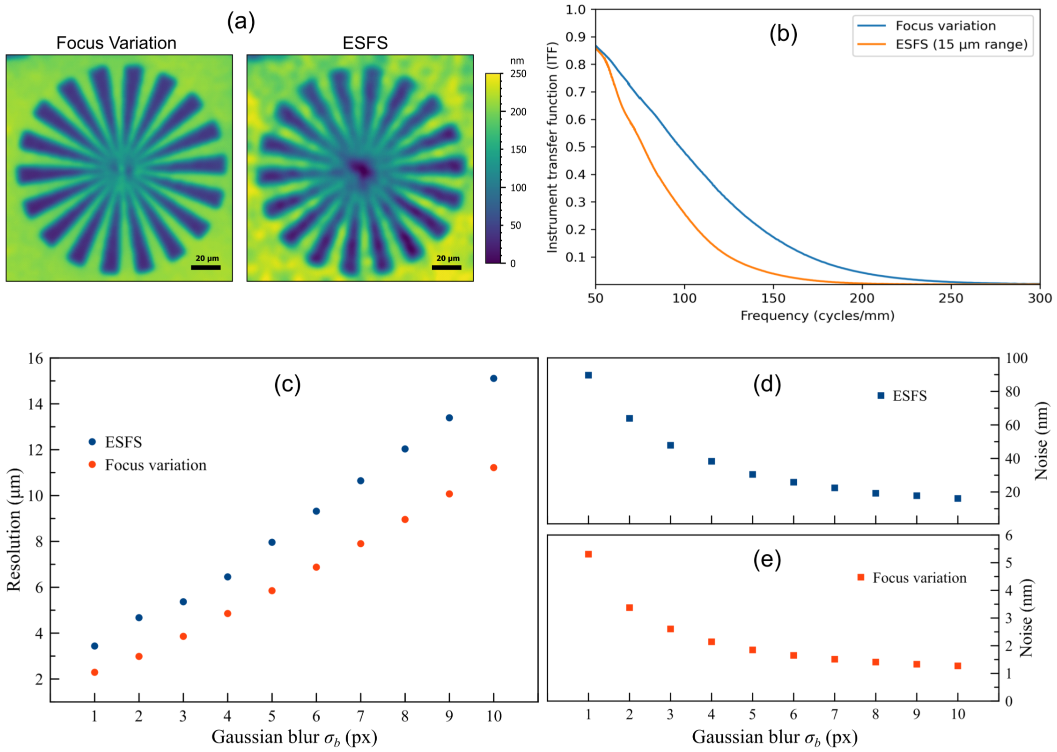

Supplementary Figure 9: Assessment of topographic resolution and system noise for ESFS and conventional focus variation. (a) Topographies of a Siemens star target acquired with a 50×/0.8NA objective, acquired using ESFS and a conventional focus variation technique. (b) Instrument Transfer Function (ITF) curves calculated from the topographies in (a); in both cases the applied Gaussian blur is $\sigma_b = 5$px. (c) Plot of the estimated topographic resolution as a function of σ_b. (d,e) Calculation of the system noise as a function of σ_b. Results for ESFS have a measurement range of 15 µm; for the plane-by-plane scanning conventional technique, the range is irrelevant.

Supplementary Figure 9 are in good agreement with the expected behaviour. Interestingly, the system noise is significantly increased in the case of ESFS compared to traditional focus variation, but the lateral resolution is only slightly sacrificed. Importantly, though, the number of images required to properly reconstruct a topography map is drastically reduced in ESFS, as widely discussed in the main text.

The second prototype is implemented as an add-on to a commercial inverted microscope (Nikon, Eclipse Ti2). A fast varifocal tuneable acoustic gradient (TAG) lens is placed at a plane conjugate to the objective pupil through a 4f configuration, which is also used to insert the illumination with structured light, as shown in Figure 3a of the main paper. A photograph of the system is shown in **Supplementary Figure 10**. The TAG lens is driven with a sinusoidal function with 20 V of amplitude and a frequency of 70 kHz.

A summary of the results of measurements on standard targets to assess the performance of both systems is shown in **Supplementary Table 3**.

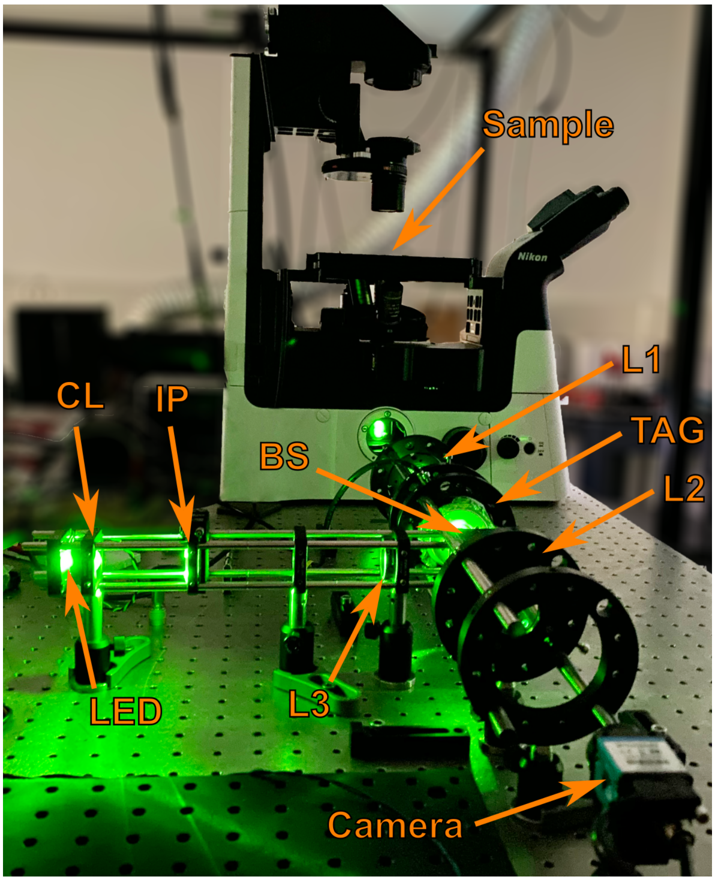

Supplementary Figure 10: Prototype of the ESFS method implemented in a commercial microscope with a fast varifocal lens. A 4f relay system is mounted on the optical port using lenses L1 and L2, to place the TAG lens optically-conjugated with the objective pupil. A beam splitter BS is used to insert illumination, which employs LED light collimated with a condenser lens CL. A transmissive Ronchi mask is placed at the image plane IP such that the pattern is projected onto the sample plane through lens L3. The optical layout is shown in Figure 3a of the main paper.

Supplementary Table 3: Accuracy evaluation on measurements of standard targets. Reference uncertainty values correspond to expanded uncertainty with factor $k = 2$.

	Specimen/measurand	Reference (µm)	Uncertainty (µm)	Measurement (µm)
1st prototype	AIR B40 - Sa	0.79	0.03	0.77
	AIR B40 - Sq	1.00	0.02	0.99
	Step Height (20µm)	21.702	0.022	21.704
2nd prototype	Step Height (0.5µm)	0.541	0.012	0.538
	Step Height (1µm)	0.926	0.012	0.929
	Step Height (2µm)	1.918	0.012	1.924

5 Supplementary references

[1] S. Zhang, "Absolute phase retrieval methods for digital fringe projection profilometry: A review," *Optics and Lasers in Engineering*, vol. 107, pp. 28–37, 2018.

[2] J. Bruning, D. Herriott, J. Gallagher, D. Rosenfeld, A. White, and D. Brangaccio, "Digital wavefront measuring interferometer for testing optical surfaces and lenses," *Applied optics*, vol. 13, pp. 2693–703, 11 1974.

[3] Q. Zhang, X. Su, L. Xiang, and X. Sun, "3-d shape measurement based on complementary gray-code light," *Optics and Lasers in Engineering*, vol. 50, no. 4, pp. 574–579, 2012. Computational Optical Measurement.
