# Supplementary material for: Fast topographic optical imaging using encoded search focal scan
Source: Nat Commun. 2024 Mar 7;15:2065. doi: 10.1038/s41467-024-46267-y (PMC10920621; doi:10.1038/s41467-024-46267-y)
Supplement: Supplementary file 3 — Description of Additional Supplementary Files [file 41467_2024_46267_MOESM3_ESM.pdf]

**Description of Additional Supplementary Files**

**Supplementary Movie 1**. ESFS-enabled topographic reconstructions of the roughness standard AIRB40 while being manually translated. The 3D profiles were acquired with prototype 2 at a rate of 67 topographies per second.

**Supplementary Movie 2**. ESFS-enabled topographic reconstructions of a MEMS membrane undergoing deformation caused by periodic electric-induced heating at 6 Hz. The 3D profiles were acquired with prototype 2 at 67 frames per second. Imaged volume 248.4 × 186.3 × 54 $\mu m^3$.

**Supplementary Movie 3**. ESFS-enabled topographic reconstructions of a MEMS membrane undergoing deformation caused by periodic electric-induced heating at 20 Hz. The 3D profiles were acquired with prototype 2 at 67 frames per second. Imaged volume 248.4 × 186.3 × 54 $\mu m^3$.
